# Two thermal performance test (TPT) datasets of a single U-tube borehole heat exchanger with inlet setpoint temperatures of 30 °C and 40 °C

**DOI:** 10.1016/j.dib.2018.08.215

**Published:** 2018-09-08

**Authors:** Wonjun Choi, Hideki Kikumoto, Ryozo Ooka

**Affiliations:** Institute of Industrial Science, The University of Tokyo, 4-6-1 Komaba, Meguro-ku, Tokyo 153-8505, Japan

## Abstract

The presented thermal performance test (TPT) datasets were related to the research article “New perspectives in thermal performance test: Cost-effective apparatus and extended data analysis” (Choi et al., 2018), where a new TPT apparatus was developed by adding a solid-state-relay and a proportional–integral–derivative (PID) controller to a thermal response test apparatus. Using the developed apparatus connected to a 50-m-long vertical ground heat exchanger, two TPTs were conducted for 144 h with inlet setpoint temperatures of 30 °C and 40 °C. The raw data were measured at 5 s intervals and consisted of the inlet, and outlet fluid temperatures, and the flow rate. The attached MATLAB script allows users to easily filter the data at user-specified time intervals. Moreover, the execution of code provides two additional quantities: heat injection rate and unit heat exchange rate. The datasets are shared for the following purposes: (1) performance comparison of various ground heat exchangers using the unit heat exchange rate (2) comparison of the control performance of a newly developed TPT apparatus, (3) validation of an analytical or numerical thermal response model, and (4) validation of a parameter estimation algorithm.

**Specifications table**

**Table:** 

Subject area	*Engineering, Building energy system, Renewable energy*
More specific subject area	*Shallow geothermal energy, Ground-source heat pump, Inverse problem*
Type of data	*Excel sheets with MATLAB code for data filtering*
How data was acquired	*Data logger: Keyence NR-600*
	* TH-08 (voltage module)*
	* HA-08 (current module)*
	*PT100 (Class A): manufactured and calibrated by Netsushin*
	*Electromagnetic flow meter: Keyence FD-M(Z)100AT*
	*Proportional–integral–derivative (PID) controller: Azbil SDC35*
	*Solid-state-relay (SSR): Mitsubishi US-N40*
	*Heater: Three plug-type resistance heaters (1, 2, and 4 kW)*
	*Pump: Iwaki Pump MD-100RM*
Data format	*Raw data*
Experimental factors	*No pretreatment was applied*
Experimental features	*Using the combination of a PID controller and an SSR, the inlet fluid temperature was controlled. Two thermal performance tests were conducted for 144 h with setpoint temperatures of* 30 °C and 40 °*C. The inlet and outlet fluid temperatures and the flow rate in both experiments were measured at 5 s intervals.*
Data source location	*Chiba City, Chiba Prefecture, Japan*
	*Latitude: 35.626462; longitude: 140.105586*
Data accessibility	*Data are available with this article*
Related research article	*New perspectives in thermal performance test: Cost-effective apparatus and extended data analysis, Energy and Buildings, 2018,*10.1016/j.enbuild.2018.08.008[1]

**Value of the data**

• The control performance of different apparatus configurations can be compared in terms of the initial rise time, overshoot, and steady-state error.
• The thermal performance of different ground heat exchanger configurations can be compared using the unit heat exchange rate.
• Data can be used to verify a new parameter estimation algorithm for obtaining GSHP design parameters (i.e., effective ground thermal conductivity and borehole thermal resistance).
• Data can be used to verify the accuracy of a numerical or analytical thermal response model because the experimental condition of a TPT is very stable by mechanical control.

## 1 Data

Thermal performance test (TPT), which has a constant inlet temperature as the experimental condition is a relatively new experimental method in the field of ground-source heat pump (GSHP). It is conducted to examine the thermal performance of a ground heat exchanger (GHE) with new geometrical configuration and material. Compared to the ground thermal response test (TRT) which is regarded as the industry standard for sizing of ground heat exchangers, TPTs are rarely conducted. This is because TPT apparatuses usually include a complex mechanical control and a hot water tank for the stable temperature control [2], [3], [4], [5], [6], [7], which increases the experimental cost.

In this context, we developed a new cost-effective TPT apparatus. It can be constructed by adding a solid-state-relay (SSR) and proportional–integral–derivative (PID) controller to an any TRT apparatus. Although the proposed apparatus has a very simple configuration, its control performance is as good as conventional TPT apparatuses. The shared two TPT datasets were obtained using the developed TPT apparatus connected to a 50-m-long borehole heat exchanger (BHE).

## 2 Experimental design, materials, and methods

### 2.1 Experimental setup and experimental condition

The BHE has a 50-m-long single U-tube heat exchanger made of high-density polyethylene and the annulus of borehole was backfilled with gravels. Table 1 presents the geometrical information of the BHE used for two TPTs. The details of the experimental setup and site information can be found in [1], [8]. Two TPTs were conducted for 144 h with inlet setpoint temperatures of 30 °C and 40 °C and they were denoted as TPT30 and TPT40, respectively. Table 2 summarizes the experimental conditions of two TPTs. The inlet and outlet fluid temperatures and the flow rate were measured at 5 s intervals. The sensors, data logger, SSR, PID controller and some components in the apparatus used for the experiments are listed in the Specifications table.

**Table 1:** Geometrical information of borehole heat exchanger.

Component	Parameter	Value [mm]
Heat exchanger (U-shaped tube)	Outer diameter	34
	Inner diameter	27
	Shank spacing	50
Borehole	Depth	50,000
	Diameter	165
Gravel (filling material)	Average grain diameter	10

**Table 2:** Experimental conditions of two thermal performance tests.

Test name	Duration [h]	Setpoint temperature [°C]	Average flow rate [l/min]	Average heat rate [kW]	Initial ground temperature [°C]
TPT30	144	30	18.19	1.98	16.8
TPT40	144	40	18.85	3.73	16.6

### 2.2 Shared TPT datasets and how to use the MATLAB code

The raw data of TPT30 and TPT40 were contained in independent Excel files with the file names “TPT30_RawData_5s.xlsx” and “TPT40_RawData_5s.xlsx,” respectively. The files can be downloaded from https://doi.org/10.1016/j.dib.2018.08.215. Each Excel sheet has six columns as below:

**Table:** 

Column 1	Column 2	Column 3	Column 4	Column 5	Column 6
Measured time [MM/DD/YYYY hh:mm:ss]	Elapsed time [s]	Inlet temperature [°C]	Outlet temperature [°C]	Mean temperature [°C]	Flow rate [l/min]
01/29/2017 13:00:03	0	16.84	16.82	16.83	17.444
01/29/2017 13:00:08	5	17.00	16.82	16.91	17.163
01/29/2017 13:00:13	10	17.42	16.82	17.12	17.131
⋮	⋮	⋮	⋮	⋮	⋮

**Fig. 1:**
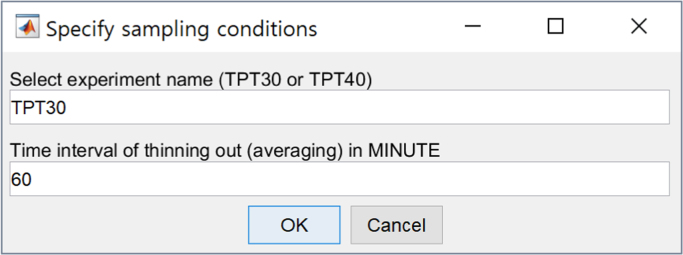
Pop-up window of the data filtering code.

The raw data can be easily filtered at the user׳s specified time intervals using the attached MATLAB script with the filename “TPT_RawDataProcess.m”. When the code is executed, the following pop-up window asks users to select an experiment name (TPT30 or TPT40) and specify filtering time intervals (Fig. 1). Note that the unit of time interval is minute. If a time interval of less than 1 min is needed (e.g., 30 s), then writing 1/2 will filter the raw data at 30 s intervals).

Three Excel files will be generated when the calculation is finished. The name of the Excel files varies depending on the user-specified conditions. For example, three Excel files will be generated if the experiment TPT30 and the time interval of 60 min are specified as the filtering conditions, and each file contains the following data:

• TPT30_instant_60min.xlsx: instantaneous data thinned out at 60 min intervals.
• TPT30_averaged_60min.xlsx: averaged data over 60 min intervals.
• TPT30_combined_60min.xlsx: instantaneous temperatures thinned out at 60 min intervals, and flow rate and heat rate average over 60 min intervals are combined.

The generated excel files have seven columns:

**Table:** 

Column 1	Column 2	Column 3	Column 4	Column 5	Column 6	Column 7
Elapsed time [s]	Inlet temperature [°C]	Outlet temperature [°C]	Mean temperature [°C]	Flow rate [l/min]	Heat rate [W/m]	Unit heat exchange rate [W/(m·K)]
0	16.84	16.82	16.83	17.44	0	0
3600	30.00	26.88	28.44	17.84	104.55	13.61
7200	30.02	27.34	28.68	17.91	71.53	6.08
⋮	⋮	⋮	⋮	⋮	⋮	⋮

The filtered file can be used according to the purpose. The combined file (e.g., "TPT30_combined_#min.xlsx") should be used if the purpose is to validate a parameter estimation algorithm or compare the thermal performance of a GHE using the unit heat exchange rate.

## 3 Usage of shared datasets

The datasets can be used for the following purposes:

1) Comparison of control performance of developed apparatus
  When a TPT apparatus with a similar configuration without a thermal buffer is developed, the difference of the control performance by implementing different control algorithms or setting PID coefficients can be compared in terms of the following aspects: (1) rise time, which is the time required to reach a setpoint temperature from an initial temperature, (2) amount of overshoot and oscillation time after the inlet temperature reaches the setpoint, and (3) amount of steady-state error after the system behavior is stabilized.
2) Benchmark for the transient thermal performance of ground heat exchangers
  How to use the TPT data has not been fully discussed, and no consensus exists among researchers. Therefore, sometimes only the heat exchange rate ($Q_{\mathrm{GHE}}=C_{f}{\dot{V}}_{f}\left(T_{f,\mathrm{in}}-T_{f,\mathrm{out}}\right)$, where $C_{f}$: volumetric heat capacity, ${\dot{V}}_{f}$: volumetric flow rate, $T_{f,\mathrm{in}}$: inlet fluid temperature; and $T_{f,\mathrm{out}}$: outlet fluid temperature) is presented as a result of the TPTs. However, in this form, comparing different TPT results is impossible because the heat exchange rate depends on the difference between the initial ground temperature and the inlet setpoint temperature. We need a normalized or dimensionless index to compare and discuss the results from different TPT settings with different GHE configurations. In this context, we suggested using the unit heat exchange rate in its W/(m·K) form or a dimensionless form [1] for comparing different GHEs installed at the same experimental site or GHEs installed at different experimental sites, respectively. Although the shared TPT datasets were obtained using a general single U-tube BHE instead of a geometrically complex GHE, they can be used as an example of the normal BHE performance.
3) Validation of a numerical or analytical thermal response model
  Unlike typical in-situ TRTs, where the temperature response is significantly disturbed by many contextual disturbances [9], [10], [11], [12], [13], TPTs can maintain the intended experimental conditions by using the mechanical control. Although in situ experiments have some uncertain factors which are hard to examine, such as the intermittent groundwater flow and the exact geometrical configuration of the GHE, the dataset of the TPT was more reliable than that of the TRT. Therefore, the TPT data can be used to validate a numerical or analytical thermal response model.
4) Validation of a parameter estimation algorithm
  Shared TPT datasets can be used for the validation purpose when a numerical or stochastic parameter estimation algorithm is developed. The estimation results of the effective ground thermal conductivity and the borehole thermal resistance using the Bayesian inference technique [14], [15] were presented in the original research article [1].

## Appendix A Supplementary material

MATLAB script for data filtering and raw data of two thermal performance tests.

## References

[1] Choi W., Kikumoto H., Ooka R. (2018). New perspectives in thermal performance test: cost-effective apparatus and extended data analysis. Energy Build..

[2] Li X., Chen Y., Chen Z., Zhao J. (2006). Thermal performances of different types of underground heat exchangers. Energy Build..

[3] Gao J., Zhang X., Liu J., Li K., Yang J. (2008). Numerical and experimental assessment of thermal performance of vertical energy piles: an application. Appl. Energy.

[4] Bi Y., Wang X., Liu Y., Zhang H., Chen L. (2009). Comprehensive exergy analysis of a ground-source heat pump system for both building heating and cooling modes. Appl. Energy.

[5] Wang H., Qi C., Du H., Gu J. (2009). Thermal performance of borehole heat exchanger under groundwater flow: a case study from Baoding. Energy Build..

[6] Park S., Lee D., Choi H.-J., Jung K., Choi H. (2015). Relative constructability and thermal performance of cast-in-place concrete energy pile: coil-type GHEX (ground heat exchanger). Energy.

[7] Yoon S., Lee S.-R., Xue J., Zosseder K., Go G.-H., Park H. (2015). Evaluation of the thermal efficiency and a cost analysis of different types of ground heat exchangers in energy piles. Energy Convers. Manag..

[8] Choi W., Ooka R. (2016). Effect of natural convection on thermal response test conducted in saturated porous formation: comparison of gravel-backfilled and cement-grouted borehole heat exchangers. Renew. Energy.

[9] Borinaga-Treviño R., Norambuena-Contreras J., Castro-Fresno D. (2015). How to correct the ambient temperature influence on the thermal response test results. Appl. Therm. Eng..

[10] Abdelaziz S.L., Olgun C.G., Martin J.R. (2015). Counterbalancing ambient interference on thermal conductivity tests for energy piles. Geothermics.

[11] Choi W., Ooka R. (2016). Effect of disturbance on thermal response test, part 1: development of disturbance analytical model, parametric study, and sensitivity analysis. Renew. Energy.

[12] Choi W., Ooka R. (2016). Effect of disturbance on thermal response test, part 2: numerical study of applicability and limitation of infinite line source model for interpretation under disturbance from outdoor environment. Renew. Energy.

[13] Hu P., Meng Q., Sun Q., Zhu N., Guan C. (2012). A method and case study of thermal response test with unstable heat rate. Energy Build..

[14] Choi W., Kikumoto H., Choudhary R., Ooka R. (2018). Bayesian inference for thermal response test parameter estimation and uncertainty assessment. Appl. Energy.

[15] Choi W., Menberg K., Kikumoto H., Heo Y., Choudhary R., Ooka R. (2018). Bayesian inference of structural error in inverse models of thermal response tests. Appl. Energy.

